# Research on Computing Resource Measurement and Routing Methods in Software Defined Computing First Network

**DOI:** 10.3390/s24041086

**Published:** 2024-02-07

**Authors:** Xiaomin Gong, Shuangyin Ren, Chunjiang Wang, Jingchao Wang

**Affiliations:** Academy of Systems Engineering, AMS, Beijing 100141, China; gongxiaomin04@163.com (X.G.);

**Keywords:** software defined network, computing first network, computing routing, computing resource measurement, reinforcement learning

## Abstract

Computing resource measurement and computing routing are essential technologies in the computing first network (CFN), serving as its foundational elements. This paper introduces a Software Defined Computing First Network (SD-CFN) architecture. Building upon this framework, a Dynamic-Static Integrated Computing Resource Measurement Mechanism (DCRMM) is proposed, incorporating methods such as the entropy weight method and K-Means clustering. The DCRMM algorithm outperforms the Maximum-closest Static Algorithm (MSA) and Maximum Closest Dynamic Algorithm (MDA) in terms of node stability, node utilization, and node matching accuracy. Additionally, a Reinforcement Learning and Software Defined Computing First Networking Routing (RSCR) algorithm is presented as a software-defined computing routing solution within the SD-CFN. RSCR introduces a knowledge plane responsible for computing routing calculations. It comprehensively considers factors such as link latency, available bandwidth, and packet loss rate. Simulation experiments conducted on the GÉANT topology demonstrate that RSCR outperforms the OSPF algorithm in terms of link latency, packet loss rate, and throughput. DCRMM and RSCR offer innovative solutions for computing resource measurement and computing routing in computing first networks.

## 1. Introduction

As artificial intelligence, big data, and other technologies continue to advance, computing-intensive and latency-sensitive tasks such as facial recognition, object detection, and autonomous driving have emerged in large numbers. This trend has driven the further development of technologies such as cloud computing and edge computing, enabling end-users to more conveniently access distributed computing resources at the edge. However, the deployment of edge computing still faces significant challenges. From a network perspective, the computing capabilities of individual nodes in edge computing scenarios are limited. Edge nodes cannot perceive each other and are unable to collaborate effectively. The challenge lies in the inability to efficiently schedule computing tasks to the optimal edge node. From a business requirements perspective, there is a need to decouple existing business application layers from the network. The application layer cannot accurately grasp the network’s status. Addressing results dominated by the application layer may not achieve optimal overall performance and could potentially lead to network load imbalance. Services may not be efficiently arranged to the best edge node, resulting in impaired business operations [1]. Therefore, it is crucial to address how to efficiently and flexibly schedule computing resources between nodes, thereby improving the utilization of computing power [2,3].

To more efficiently utilize ubiquitous distributed computing resources and expedite the processing of computing tasks, the convergence of computing and networking has become imminent. With the impetus from telecom operators and equipment vendors, the Computing First Networking (CFN) paradigm has emerged [4,5]. CFN represents a new paradigm of cloud-network integration, aiming to achieve the interconnection and coordinated scheduling of ubiquitous distributed computing, storage, and other resources through the network. This enables on-demand and real-time invocation of massive computing and storage resources, facilitating global optimization and efficient utilization of computing and network resources. As businesses transition to the cloud and the consumer internet evolves into the industrial internet, the convergence of computing and networking has become a pivotal force in the digital transformation of social and economic activities.

The CFN proposes a new network architecture and protocols, unifying the scheduling of computing and network resources, enabling global optimization and collaborative scheduling of these resources. Existing CFN architectures can be broadly categorized into two types: centralized architecture and distributed architecture. The centralized architecture, often combined with Software Defined Networking (SDN), separates the data plane from the control plane, allowing the controller to have a global view of computing and network resources for routing computation and decision-making. The distributed architecture achieves the synchronization of computing and network resource information through information exchanges between adjacent routing nodes, with each routing node handling forwarding and control decisions. Whether centralized or distributed, achieving comprehensive scheduling of computing resources across the entire network requires a routing algorithm that schedules CFN information. Similarly, the prerequisite for routing implementation is the establishment of a unified computing resource measurement and modeling mechanism.

This paper introduces a centralized computing first network architecture, namely the Software Defined Computing First Network (SD-CFN). Leveraging the advantages of control-plane separation and centralized control, this architecture forms a global view of the computing resources and networking resources. Additionally, based on this architecture, we combine methods such as entropy weight and K-Means clustering to construct a Dynamic-Static Integrated Computing Resource Measurement Mechanism (DCRMM). According to the measurement scheme and task requirements, this mechanism identifies the most suitable computing nodes for the computation. Simultaneously, we augment SDN with a knowledge plane and design a Reinforcement Learning and Software Defined Compute First Networking Routing (RSCR) algorithm within this plane. This algorithm, utilizing interactions with the environment, RL intelligence, and the global computing and networking view provided by SDN, considers factors such as link latency, bandwidth, packet loss rate, and computing resource distribution. After finding appropriate computing nodes, it rapidly calculates the computing route from the task initiator to the target computing node. Simulation results using real traffic demonstrate that RSCR outperforms traditional OSPF algorithms significantly in terms of link throughput, latency, and packet loss rate.

This article makes two main contributions:

(1) Proposes a dynamic-static integrated computing resource measurement modeling mechanism.
(2) Proposes an RL-based proactive computing routing algorithm in the SD-CFN environment.

The rest of the paper is organized as follows: Section 2 introduces related work. Section 3 provides a detailed explanation of RSCR. Section 4 details DCRMM. Section 5 introduces the RSCR routing algorithm. Section 6 analyzes the simulation results of the RSCR algorithm. The final section concludes the paper and suggests future directions for research.

## 2. Related Work

Routing is a fundamental function in the Internet, responsible for forwarding data packets from source to destination addresses. It plays a crucial role in ensuring Quality of Service (QoS) [6]. Traditional routing strategies, like Open Shortest Path First (OSPF) routing [7], may lead to issues such as network congestion and low link utilization. Compared to optimal routing methods, traditional approaches may exhibit performance differences of up to 5000 times [8]. Routing optimization has been a topic of in-depth research [9]. Existing solutions include those based on analytical optimization [10] and those leveraging machine learning [11,12] and deep learning [13,14]. Papers [9,15,16,17] also proposed various SDN routing optimization methods. In this section, we review SDN routing optimization work based on Reinforcement Learning (RL) and Deep Reinforcement Learning (DRL). Unlike model-based routing optimization algorithms, RL-based methods are model-free. Moreover, while machine learning algorithms require dataset labeling and training, RL algorithms directly interact with the network environment to learn strategies.

The key application of reinforcement learning in routing problems focuses on modeling the forwarding process using the Markov Decision Process (MDP). This process involves setting appropriate states, actions, and reward functions based on specific network scenarios. The intelligent agent adapts to various dynamic changes through interaction with the environment [18]. RL can play a crucial role in SDN routing optimization [19]. As early as 1993, Boyan et al. [20] proposed using the Q-routing algorithm to avoid network congestion, marking the first academic use of reinforcement learning for routing optimization. Although this algorithm was not applied in an SDN architecture, it provided a new reference direction for subsequent routing optimization algorithms. Lin et al. [21] introduced multi-layer SDN QoS-aware adaptive routing, combining the work of Hassas [22] and McCauley [23], introducing a distributed control plane architecture, and achieving a reliable SDN infrastructure with minimal signal latency. Rischke et al. [24] proposed the QR-SDN algorithm to create multiple paths between source and destination addresses, maintaining flow integrity using Q-Learning, and employing the softmax function as an exploration-exploitation strategy. However, this algorithm can only guarantee lower flow latency than Shortest Path First (SPF) in small networks.

Houda et al. [25] used the Q-Learning algorithm to address the delay minimization problem in multipath SDN networks. They employed two different exploration strategies, namely ϵ-greedy, and softmax, and evaluated the performance of the Q-Learning algorithm based on average flow latency and convergence time for different load levels. Casas-Velasco et al. [26] proposed the RSIR algorithm, which defined an active routing algorithm based on link-state, exhibiting lower overall latency and better load balancing compared to the traditional Dijkstra algorithm. Building on this work, they also introduced Deep Reinforcement Learning (DRL) and defined the DRSIR algorithm [27] in SDN. DRSIR considers path state indicators to generate active, efficient, and intelligent routing to adapt to dynamic traffic changes. The evaluation of DRSIR was conducted through simulation using real and synthetic traffic matrices. Chen et al. [9] also introduced DRL into the routing process and proposed the RL-Routing algorithm to address Traffic Engineering (TE) issues in SDN regarding throughput and latency. They performed comprehensive experiments based on Fattree, NSFNet, and ARPANet topologies, demonstrating that experience-driven artificial intelligence has advantages over traditional algorithms in solving TE problems. Yu et al. [28] proposed a DDPG routing optimization solution, which, unlike the previous Q-table-based mechanism, saves time and storage by using a neural network. Zhao et al. [29] positioned the optimal multicast routing problem in SDN as a multi-objective optimization problem. They designed an intelligent multicast routing algorithm, DRL-M4MR, to construct a multicast tree in SDN. After DRL-M4MR agent training, the SDN controller installs multicast flow entries by reverse traversing the multicast tree to SDN switches, achieving intelligent multicast routing.

Currently, RL routing optimization algorithms based on the SDN architecture are primarily centered around Q-Learning. This paper also adopts the Q-Learning algorithm to design the reward function and adjust the strategy based on link available bandwidth, latency, and packet loss rate. The proposed RSCR algorithm exhibits superior performance compared to the traditional OSPF algorithm.

## 3. DCRMM

This section primarily introduces the unified modeling and measurement of dynamic and static performance indicators of computing nodes by DCRMM and explains in detail how to select the optimal computing node based on the measurement results. The dynamic performance indicators of computing nodes refer to the node’s idle CPU cores (cores) $c_{f}$, idle memory (GB) $m_{f}$, and idle storage (GB) $s_{f}$, while static performance indicators refer to the node’s total CPU cores (cores) $c_{t}$, total memory (GB) $m_{t}$, and total storage (GB) $s_{t}$. The workflow of DCRMM mainly consists of the following steps: a. Use the entropy weight method to calculate the static performance baseline score of computing nodes; b. Segment the baseline scores using the K-Means clustering algorithm; c. Using the entropy weight method to calculate the weights of dynamic indicators, assigning these weights to various indicators of computing tasks (i.e., the required CPU cores (cores) $t_{c}$, memory (GB) $t_{m}$, storage (GB) $t_{s}$ for computing tasks), scoring the computing tasks based on these weights, and classifying them into the corresponding static performance score intervals according to the scores. d. Within the qualifying interval, calculate the Euclidean distance between the dynamic performance of computing nodes and the required performance for computing tasks, and the node with the shortest distance is considered the optimal computing node. The reasons for using the entropy weight method are the following two points: a. The basis for determining the weight of the entropy weight method comes from the data, which are highly objective and reduce the impact of subjectivity on the decision-making results. b. The calculation logic of the entropy weight method is simple and clear, has good operability, and is easy to implement. To prevent a single indicator from occupying a high weight for a long time in the actual application process, we can reasonably set the update cycle according to environmental changes to cope with the dynamic updates of different indicators of the computing power node as the computing tasks are processed. Compared to traditional algorithms such as the Maximum-closest Static Algorithm (MSA), which considers only static indicators, and the Maximum closest dynamic algorithm (MDA), which considers only dynamic indicators, DCRMM performs better in terms of stability, node utilization, and accuracy in node selection.

### 3.1. Calculating the Basic Score

Firstly, the entropy weight method [30] is employed to analyze the static indicators of nodes, yielding the foundational performance scores. Drawing inspiration from the concept of information entropy, the entropy weight method’s greatest advantage lies in mitigating the influence of human factors on indicator weights, ensuring that the results of comprehensive evaluations are more objective [31].

Assuming there are *n* evaluation indicators and *m* objects, the raw data can be represented as a matrix *X*:

(1) $$\begin{array}{c} X=\left[\begin{array}{ccc} x_{11} & \cdots & x_{1n}\\ \vdots & \ddots & \vdots \\ x_{m1} & \cdots & x_{mn} \end{array}\right] \end{array}$$

Here, $x_{ij}$ represents the numerical value of the *j*-th indicator for the *i*-th object. Since various evaluation indicators may have different dimensions, normalization is required before calculation to ensure that the results fall within the [0,1] range. Therefore, we normalize the matrix *X* column-wise, where $X_{i}$ represents a value in the column where the standardization occurs. The normalization function is given by:

(2) $$\begin{array}{c} x_{ij}^{\prime }=\frac{x_{ij}-min\left(X_{i}\right)}{max\left(X_{i}\right)-min\left(X_{i}\right)} \end{array}$$

To avoid the impact of zero in the data on subsequent calculations, it is common to apply an offset to the data. The normalized data can be represented as a matrix $X^{\prime }$:

(3) $$\begin{array}{c} X^{\prime }=\left[\begin{array}{ccc} x_{11}^{\prime } & \cdots & x_{1n}^{\prime }\\ \vdots & \ddots & \vdots \\ x_{m1}^{\prime } & \cdots & x_{mn}^{\prime } \end{array}\right] \end{array}$$

Let the weight of the *j*-th indicator in the *i*-th object be denoted as $p_{ij}$:

(4) $$\begin{array}{c} p_{ij}=\frac{x_{ij}^{\prime }}{\sum_{i=1}^{n}x_{ij}^{\prime }},\left(0⩽p_{ij}⩽1\right) \end{array}$$

Subsequently, the weight matrix *P* for the original data is obtained as follows:

(5) $$\begin{array}{c} P=\left[\begin{array}{ccc} p_{11} & \cdots & p_{1n}\\ \vdots & \ddots & \vdots \\ p_{m1} & \cdots & p_{mn} \end{array}\right] \end{array}$$

In the entropy weight method, the entropy value of the *j*-th indicator is given by [31]:

(6) $$\begin{array}{c} e_{j}=-\frac{1}{\ln m}\;\sum_{i=1}^{n}p_{ij}\;\ln p_{ij},\left(0⩽e_{j}⩽1\right) \end{array}$$

The entropy value $e_{j}$ indicates that the larger its numerical value, the higher the differentiation degree of the *j*-th indicator, suggesting the derivation of more information. Therefore, a higher weight should be assigned to this indicator. Thus, the calculation method for the weight $w_{j}$ is given by:

(7) $$\begin{array}{c} w_{j}=\frac{1-e_{j}}{\sum_{j=1}^{m}\left(1-e_{j}\right)} \end{array}$$

Therefore, the comprehensive score $F_{i}$ for the *i*-th object can be represented as:

(8) $$\begin{array}{c} F_{i}=\sum_{j=1}^{m}p_{ij}w_{j} \end{array}$$

The static performance indicators of the computing nodes can be defined as a triplet $I=(c_{t},m_{t},s_{t})$, and through the entropy weight method, the weight triplet corresponding to each indicator is obtained $W=(w_{1},w_{2},w_{3})$. Since only numerical calculations are considered when calculating the base score, the impact of different units can be ignored. The basic score $S_{n}$ for the static performance of the computing node can be represented as:

(9) $$\begin{array}{c} S_{n}=c_{t}\;\;\;w_{1}+m_{t}\;\;\;w_{2}+s_{t}\;\;\;w_{3} \end{array}$$

$S_{n}$ represents the static performance score of the computing node. A higher value indicates that the node has stronger basic performance, making it more capable of handling computing tasks with high demands for computation, storage, and memory performance.

### 3.2. Segmenting the Basic Score

Next, the K-Means clustering algorithm is applied to segment the basic score. The K-Means algorithm is a commonly used clustering method that divides data points into different clusters or groups, so that similar data points are close to each other, while dissimilar data points are farther apart. This algorithm is an unsupervised learning method, as it does not require prior knowledge of the categories of data points but automatically identifies and groups them. By using the K-Means algorithm, nodes are divided into levels according to performance, and tasks are divided into corresponding performance nodes according to computing task resource requirements, which can reduce the amount of calculation of Euclidean distance.

Typically, the K-Means algorithm can be broken down into the following six steps:

- **Inputs:** Input the dataset $S_{n}=\left\{S_{1},S_{2},\ldots ,S_{i},\ldots ,S_{m}\right\}$ ($S_{n}$ represents the set of all basic scores) and the maximum number of iterations max_interations are provided.
- **Initialization:** Define the number of clusters *K* (In this article, *K* = 3), and initialize the cluster centroids $C^{\left(0\right)}=\left\{C_{1}^{\left(0\right)},C_{2}^{\left(0\right)},\ldots ,C_{K}^{\left(0\right)}\right\}$. The initial cluster of centroids can be randomly chosen.
- **Assignment:** For each data point $S_{i}$, calculate its distance to each cluster centroid $C_{j}$, typically using the Euclidean distance measured by the formula:
  (10) $$\begin{array}{c} d\left(S_{i},C_{j}\right)=\sqrt{\sum_{k=1}^{n}(S_{ik}-C_{jk}{)}^{2}} \end{array}$$
  Subsequently, assign each data point $S_{i}$ to the cluster with the nearest centroid $C_{j}$ where *j* is determined by the following formula:
  (11) $$\begin{array}{c} j=arg\;\;min_{j}\;\;d\left(S_{i},C_{j}\right) \end{array}$$
- **Update Centroids:** For each cluster *j*, calculate the new cluster center $C_{j}^{\left(t+1\right)}$ as the mean of all data points in that cluster:
  (12) $$\begin{array}{c} C_{j}^{\left(t+1\right)}=\frac{1}{\left|C_{j}\right|}\sum_{S_{i}\in C_{j}}x_{i} \end{array}$$
  where $\left|C_{j}\right|$ represents the number of data points in the cluster *j*, and represents the iteration number.
- **Convergence Check:** Usually, K-means iterates until a stopping condition is met, such as when the cluster centers no longer undergo significant changes (or changes are below a certain convergence threshold ϵ), or when the maximum number of iterations max_interations is reached. This can be expressed as:
  (13) $$\begin{array}{c} ∥C_{j}^{\left(t+1\right)}-C_{j}^{\left(t\right)}∥<\epsilon \end{array}$$
- **Outputs:** The final result of the K-Means algorithm includes the ultimate cluster centers $C^{\left(t\right)}=\left\{C_{1}^{\left(t\right)},C_{2}^{\left(t\right)},\ldots ,C_{K}^{\left(t\right)}\right\}$ and the set of data points assigned to each cluster $Z_{i}\subset \left\{Z_{1},Z_{2},\ldots ,Z_{n}\right\}$.

Finally, all basic scores are divided into K categories $C^{\left(t\right)}=\left\{C_{1}^{\left(t\right)},C_{2}^{\left(t\right)},\ldots ,C_{K}^{\left(t\right)}\right\}$, and each category has the following scores $Z_{i}\subset \left\{Z_{1},Z_{2},\ldots ,Z_{n}\right\}$.

### 3.3. Matching the Cluster

Subsequently, evaluate the resource demand indicators of the computing tasks, identifying the cluster of static performance scores corresponding to their scores. The resource demand of a computing task can be defined as a triplet $T=(t_{c},t_{m},t_{s})$. The score of the tasks can be calculated using the weighted triplet $W^{\prime }=\left(w_{1}^{\prime },w_{2}^{\prime },w_{3}^{\prime }\right)$ obtained during the computation of the dynamic performance score of the computing nodes. Therefore, the score of the computing task can be expressed as $S_{t}$:

(14) $$\begin{array}{c} S_{t}=t_{c}\;\;\;w_{1}^{\prime }+t_{m}\;\;\;w_{2}^{\prime }+t_{s}\;\;\;w_{3}^{\prime } \end{array}$$

If $Z_{i}min<S_{t}<Z_{i}max$, then the matching of computing nodes only needs to be performed within the set $Z_{i}$ of computing tasks. This reduces the number of nodes to be matched, improving the matching time.

### 3.4. Selecting a Computing Node

Compute the Euclidean distance between the dynamic performance of each computing node within the interval and the computing task’s resource requirements to find the most suitable computing node. To avoid selecting a computing node based on a single Euclidean distance that cannot execute the computing task, it is also necessary to exclude computing nodes in the set $Z_{i}$ that do not meet the requirements. Suppose the static performance indicator triplets of a computing node, denoted as $I=(c_{t},m_{t},s_{t})$, have at least one indicator not less than the corresponding triplet in the computing task’s resource triplets $T=(t_{c},t_{m},t_{s})$. In that case, the new matching interval can be defined as $Z_{i}^{\prime }=\left\{z_{i}|z_{i}.c_{t}⩾t_{c},z_{i}.m_{t}⩾t_{m},z_{i}.s_{t}⩾t_{s}\right\}$.

The dynamic performance of a computing node can be defined as a triplet $J=(c_{f},m_{f},s_{f})$. Considering that the dynamic performance values are continuously changing, if we denote a computing task as Task and a computing node as Node, the smaller the distance between Task and Node, the smaller the difference between them. In other words, the computing node (Node) better satisfies the conditions of the computational task (Task). Therefore, this paper adopts the n-dimensional Euclidean distance [32] as a measurement standard to assess the dynamic performance and select computing nodes suitable for computational tasks.

Suppose the computational task’s resource requirements are denoted as $T_{i}=\left(t_{ci},t_{mi},t_{si}\right)$ and the dynamic performance of the computing node as $J_{i}=\left(c_{fi},m_{fi},s_{fi}\right)$. Due to different dimensions, normalization is necessary for both $T_{i}$ and $J_{i}$. Let us denote the normalized versions as $T_{i}^{\prime }$ and $J_{i}^{\prime }$ using the formula (2). The distance between them can then be expressed as:

(15) $$\begin{array}{c} d\left(T^{\prime },J^{\prime }\right)=\sqrt{\sum_{i=1}^{n}{\left(T_{i}^{\prime }-J_{i}^{\prime }\right)}^{2}},\left(n=3\right) \end{array}$$

Calculate the set of distances $Z_{i}^{\prime }$ between all computing nodes in *D* and the computational task. The minimum distance $D_{min}$ indicates that the resource requirements of computing tasks are closest to the performance of this computing node. Therefore, this node is the best computing node that matches the resource requirements of the computational task.

## 4. RSCR

This chapter provides an overview of RSCR and introduces the functions of its major modules.

### 4.1. Overview

As early as 2003, Clark et al. [33] proposed the addition of a knowledge plane to achieve intelligence in networks. Subsequently, Mestres et al. [34] built on this concept and introduced the idea of a Knowledge-Defined Network (KDN). RSCR, on the foundation of SD-CFN, incorporates a knowledge plane where RL is integrated to achieve intelligent computing routing.

RSCR utilizes available bandwidth, delay, and packet loss rate as features for the RL process. Combined with the optimal computing node identified in Section 3, it computes the route from the computing request source to the optimal computing node. RSCR, leveraging the global view of SDN, can dynamically adjust routes based on changing traffic patterns. Furthermore, unlike traditional routing that relies on protocols such as RIP, OSPF, and BGP [35], RSCR, as described in paper [36,37,38], integrates with SDN using new routing protocols. In SD-CFN, timely and reliable communication is also crucial. Reference [39] evaluates the timeliness of drone-assisted networks, and we can introduce protocols centered on the Age of Information (AoI) for further research in the future. Regarding SD-CFN architecture communication security, we can try to introduce blockchain technology to ensure data security and privacy. Wang et al. [40] proposed a blockchain-assisted distributed access control scheme for drone computing networks, which can enable drones to autonomously manage their identities, attributes, and access policies. Referring to this solution, SD-CFN can be provided with higher efficiency and fault tolerance, making reliable computing services possible.

**Figure 1:**
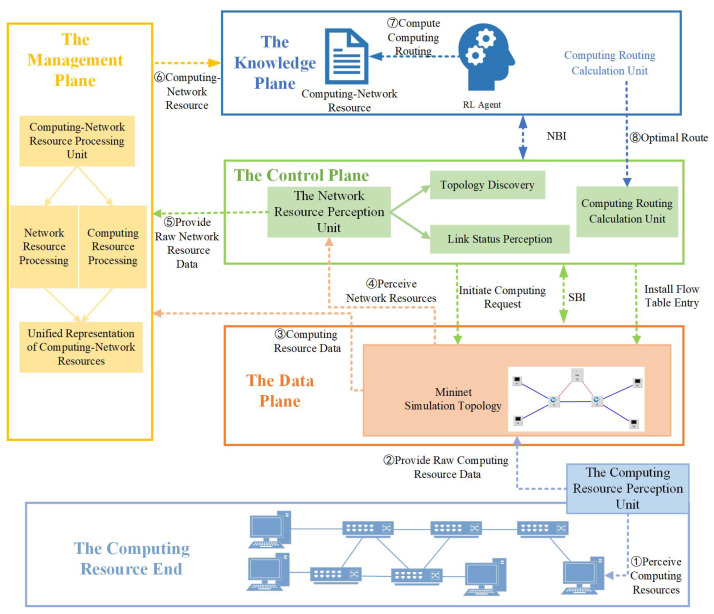
RSCR Architecture.

As shown in Figure 1, a detailed explanation of RSCR is provided below: (1) The Computing Resource Perception Unit periodically perceives the computing resources of the computing devices on the computing resource end in the data plane. Computing resources include CPU, memory, and storage resources. It is important to note that the computing resource end is composed of a Kubernetes cluster responsible for providing computing resource data. (2) The Network Resource Perception Unit periodically queries the data plane to collect raw data on the state of the network topology. (3) The Management Plane retrieves computing resource data to segment computing nodes. (4) The Management Plane retrieves network resource data to calculate and store link state information. (5) The Knowledge Plane retrieves information about the overall computing-network resources from the management plane. (6) RL Agent explores all possible routes between each source-destination node pair and computes the optimal route based on link states. (7) The Knowledge Plane stores all routing information. (8) The Control Plane retrieves routing information before the computing task arrives and proactively installs the optimal path between the computing request source and the optimal computing node in the form of flow tables on the switches.

### 4.2. Architecture

**Figure 2:**
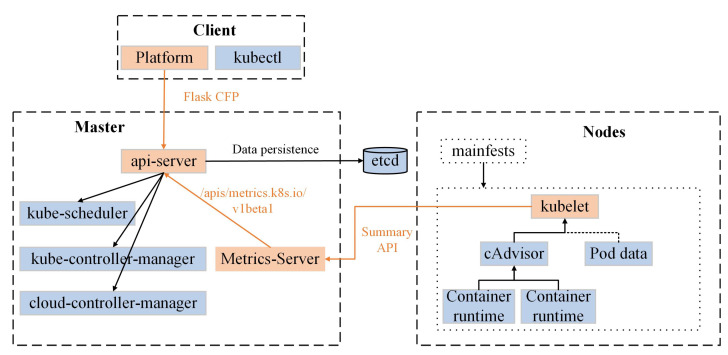
Computing Resource Perception Process.

(1) *The Data Plane:* The data plane is a collection of all infrastructure, including end devices, forwarding devices, and the links connecting them. Responsible for the actual processing and forwarding of data packets, it utilizes the programmability and real-time response of flow table rules. It executes instructions from the control plane, implements RSCR routing policies, and responds to the periodic transmission of computing resources and network resource information by the computing resource perception unit and network resource awareness unit.

(2) *The Control Plane:* The control plane is a core component of the architecture, responsible for formulating network policies and dynamic management, constructing a global view of the data plane. Separated from the data plane, it uses a centralized controller to manage and adjust flow table rules of network devices in real-time, responding to the requirements of different applications and services to optimize network performance, security, and availability. This plane mainly consists of two modules: the network resource awareness unit and the computing route deployment unit.

(3) *The Management Plane:* The management plane is a crucial component of the SDN architecture, responsible for centralized network management and control. It provides network administrators with an abstract way to define network policies, configure devices, monitor network performance, and apply intelligent decision-making, achieving flexibility, automation, and intelligent management of the network. The management plane collaborates with the data plane, where the computing unit processes collected raw data on computing resources and network resources to segment computing nodes and calculate available bandwidth, latency, and packet loss rates. These data describe the computational capabilities and network status of computing nodes.

(4) *The Knowledge Plane:* The knowledge plane is a core component of RSCR, designed to integrate and manage widely distributed knowledge sources to support intelligent decision-making and reasoning. It provides functions for the storage, retrieval, inference, and application of knowledge, enabling the system to automatically analyze and understand information for better meeting user needs and supporting intelligent decision-making. This plane, through the management plane, processes well-handled computing-network resource data and computing task requests proposed by the data plane to calculate and install the optimal route. The deployment of the knowledge plane is highly flexible; it can be deployed on top of the control plane, similar to the application plane in standardized SDN [41], or deployed separately [34]. RSCR opts for a separate deployment of the knowledge plane to avoid overloading the control plane.

(5) *The Computing Resource Perception Unit:* This unit is responsible for perceiving the computing resources of each computing node in the computing resource end and sending the perceived raw data to the computing-network resource processing unit of the management plane. The computing resource perception process is illustrated in Figure 2, involving three main components: Client, Master, and Cluster. The Master is in charge of the entire Kubernetes cluster, composed mainly of API-Server, kube-scheduler, kube-controller-manager, and cloud-controller-manager. Additionally, the Metrics-Server is deployed on the Master. Nodes receive instructions from the Master to carry out tasks. It is noteworthy that the Metrics-Server is not part of the API-server; it is independently deployed based on the Aggregator plugin mechanism, providing services uniformly to the API-server. By accessing the /api/metrics.k8s.io/v1beta1 interface exposed by Metrics-Server, periodic data can be obtained. These data are collected from the kubelet’s Summary API and include both cAdvisor’s monitoring data and kubelet’s summary information. Through processing the collected information, extracting computing resources is performed to achieve computing resource awareness.

(6) Network Resource Perception Unit: This unit is divided into two functions: Topology Discovery and Link Status Perception. The Topology Discovery module sends request messages to forwarding devices (switches) in the data plane, and the forwarding devices respond by sending feature information such as ID, port count, and port status. Based on the received messages, this module associates each port of each switch with neighboring switch ports and hosts connected to each switch port, thereby inferring the network topology. The Link Status Perception module is mainly responsible for perceiving fundamental data on link status in terms of computing delay ($d_{link}$), instantaneous throughput ($bwu_{link}$), and packet loss rate ($l_{link}$).

(7) Computing-Network Resource Processing Unit: This unit mainly consists of two modules, namely Network Resource Processing and Computing Resource Processing. Network Resource Processing is responsible for processing the collected raw network data, calculating available bandwidth ($bwa_{link}$), delay ($d_{link}$), and packet loss ($l_{link}$). The controller periodically sends Port-Stats-Request messages to specified switches at each port. It retrieves the sent byte count ($bt_{1}$), received byte count ($bt_{2}$), and port lifetime (time difference between sending and receiving data) δt from the Port-Stats-Reply messages from the switches. With this information, it can calculate the instantaneous throughput $bwu_{link}=\frac{b_{t2}-b_{t1}}{\Delta t}$. Therefore, the available bandwidth of the link ($bwa_{link}$) is represented as the difference between link capacity ($cap_{link}$) and instantaneous throughput can be represented as $bwa_{link}=cap_{link}-bwu_{link}$. The link’s instantaneous packet loss rate ($l_{link}$) can be represented as $l_{link}=\frac{b_{t1}-b_{t2}}{b_{t1}}$.

The calculation of link latency follows the method described in reference [42], which adheres to the Link Layer Discovery Protocol (LLDP) [43] and the OpenFlow protocol [44]. The SDN controller $c_{0}$ sends LLDP messages, and the messages traverse the path $c_{0}-s_{i}-s_{j}-c_{0}$, where $s_{i}$, $s_{j}$ represent the switches connected by the link $\left(s_{i},s_{j}\right)$. The time difference between the LLDP message transmission and reception, denoted as $d_{lldp_{cij}}$, estimates the time it takes for the message from $c_{0}$ to port $s_{i}$. This estimation considers half of the time for the transmission and reception of OpenFlow echo_request and echo_reply messages sent from $c_{0}$ to $s_{i}$. A similar process is used to estimate the time the message takes from $s_{j}$ to $c_{0}$. Therefore, the instantaneous latency of the link $\left(s_{i},s_{j}\right)$, denoted as $d_{link}$, can be represented as $d_{link}=d_{s_{i}-s_{j}}=d_{lldp_{cij}}-d_{c_{0}-s_{i}}-d_{s_{j}-c_{0}}$.

Computing-Network Resource Processing involves segmenting compute nodes based on their computing capabilities according to the DCRMM algorithm, using the collected compute resource data.

(8) Computing Routing Calculation Unit: Analyzing the current network link status and compute distribution based on computing-network resource data, coupled with the compute resource requirements of computing tasks, this unit calculates the optimal routing. The specific calculation method will be detailed in Section 5.

(9) Computing Routing Deployment Unit: This unit proactively writes flow table entries into the switches in the network through SDN applications, guiding the processing and forwarding of traffic. This process is typically accomplished using the Southbound Interface (SBI). By installing flow table entries on switches, the SDN controller can precisely define how to handle specific types of traffic, including routing, filtering, forwarding, or modification. The installation of flow table entries affects the traffic forwarding in the data plane [45]. Effective installation of flow table entries can optimize network performance and implement various network policies, but incorrect installation may lead to network failures or performance issues.

## 5. SD-CFN Routing

SD-CFN architecture’s routing adopts the Q-Learning algorithm. Q-learning is a classic reinforcement learning algorithm used to solve Markov Decision Process (MDP) problems. Its core idea is to learn a state-action value function $Q_{t}\left(S_{t},A_{t}\right)$, which evaluates the long-term expected return for taking a specific action in a given state. This algorithm is a model-free algorithm, so it does not require knowledge of the potential returns for taking specific actions in specific states [46]. For the state-action value function $Q_{t}\left(S_{t},A_{t}\right)$, its update rule can be expressed as:

(16) $$\begin{array}{c} Q_{t}\left(S_{t},A_{t}\right)=\left(1-\alpha \right)\;Q_{t}\left(S_{t},A_{t}\right)+\alpha \;\left(r+\gamma \;max_{A_{t+1}}Q_{t+1}\left(S_{t+1},A_{t+1}\right)\right) \end{array}$$

Here, $Q_{t}\left(S_{t},A_{t}\right)$ is the estimated value (Q-value) of selecting action $A_{t}$ in state $S_{t}$, α is the learning rate, *r* is the immediate reward obtained after selecting action $A_{t}$ in state $S_{t}$, γ is the discount factor, and $max_{A_{t+1}}Q_{t+1}\left(S_{t+1},A_{t+1}\right)$ represents the maximum estimated value after selecting the optimal action $A_{t+1}$ in the next state $S_{t+1}$.

In this paper, the learning of the agent involves a series of steps transitioning from the initial state to the target state (the computing task requester and the target computing node). Each step includes selecting and executing actions, changing the state (transitioning from one state to another), and receiving rewards. The updated Q-function values are based on the fundamental rewards obtained by taking action in the state, providing the optimal reward. Next, we will provide a detailed introduction to the RL agent and the RL routing algorithm.

### 5.1. Reinforcement Learning Agent

(1) State Space (*S*): Each state in the state space corresponds to a forwarding device in the data plane, specifically referring to SDN switches in this context. State transitions correspond to links connecting two switches. Therefore, the size of the state space is equal to the number of switches in the network topology.

(2) Action Space (*A*): It corresponds to the set of all actions taken for all states within the state space *A*. For a given state $s_{i}\in S$, the reinforcement learning agent can take any action, and each action leads to a transition from state $s_{i}$ to one of its neighboring states. The neighboring states of state $s_{i}$ correspond to the adjacent switches related to the state $s_{i}$. Therefore, the number of actions for state $s_{i}$ is equal to its degree $dr(s_{i})$, which is the number of switches connected to that state. The size of the action space *A* can be defined as $\left|A\right|\equiv \sum_{s_{i}\in S}dr(s_{i})$.

(3) Reward Function: The reward is inversely proportional to the available bandwidth $bwa_{link}$, and directly proportional to the delay $d_{link}$ and packet loss rate $l_{link}$. $\beta_{1},\beta_{2},\beta_{3}\in \left[0,1\right]$ are tunable parameters, allowing for different weights to be set for different features.

(17) $$\begin{array}{c} R=\beta_{1}\;\frac{1}{bwa_{link}}+\beta_{2}\;d_{link}+\beta_{3}\;l_{link} \end{array}$$

Due to the different dimensions of bandwidth $bwa_{link}$, latency $d_{link}$, and packet loss rate $l_{link}$, it is necessary to normalize using Equation (2). Equation (18) provides the normalized reward function:

(18) $$\begin{array}{c} R^{\prime }=\beta_{1}\;\frac{1}{bwa_{link}^{\prime }}+\beta_{2}\;d_{link}^{\prime }+\beta_{3}\;l_{link}^{\prime } \end{array}$$

(4) Optimal Policy: The goal of the optimal policy is to minimize the reward values in the Q-Learning routing process. In this way, the agent can choose paths with high available bandwidth, low latency, and a small packet loss rate during the routing process. The intelligent agent approximates the optimal Q-function by accessing all action-state pairs. It updates and stores Q-values in the Q-table, which is used to find the optimal path from the computing request source to the target computing node. The Q-value is a measure of the overall expected reward when the reinforcement learning agent is in state $S_{t}$ and takes action $A_{t}$. The reinforcement learning agent updates the Q-value using Equation (19). In Equation (19), the updated Q-value ($Q_{t+1}$) depends on the previous value $Q_{t}$, and it is influenced by the result $\left(S_{t},A_{t},R_{t},S_{t+1}\right)$ and α. $R_{t}$ is the reward at time *t*, and α determines the weight relationship between the newly acquired information and previous information. α=0 would make the reinforcement learning agent unable to learn from the latest $\left(S_{t},A_{t}\right)$ pair, while α=1 allows the agent to retain learned information by considering the immediate reward *R_t_* for the $\left(S_{t},A_{t}\right)$ pair.

(19) $$\begin{array}{c} Q_{t+1}\left(S_{t+1},A_{t+1}\right)=Q_{t}\left(S_{t},A_{t}\right)+\alpha \;\left[R_{t}+min_{A}Q_{t}\left(S_{t+1},A\right)-Q_{t}\left(S_{t},A_{t}\right)\right] \end{array}$$

(5) Exploration and Exploitation Strategy: In Q-Learning, there is a trade-off between choosing the expected optimal action (exploitation) and choosing different actions in the hope of obtaining greater rewards in the future (exploration) [47]. This paper adopts the ϵ-greedy exploration and exploitation method. ϵ-greedy uses ϵ∈[0,1] as a tunable parameter, allowing the intelligent agent to exploit with probability pr=ϵ and explore with probability pr=1−ϵ. Therefore, this parameter determines the degree to which the reinforcement learning agent explores and exploits during the learning process. The agent uses Equation (20) to choose the next action for a specific state. At each step, it generates a random value x∈[0,1]. If x<ϵ, the agent exploits; otherwise, the agent explores.

(20) $$\begin{array}{c} A=\left\{\begin{array}{c} min_{A}Q_{t}\left(S_{t},A\right),x<\epsilon \\ random\;\;action,otherwise \end{array}\right. \end{array}$$

### 5.2. Routing Algorithm

This routing algorithm implements a learning process for finding the optimal paths for all node pairs in the data plane. The input data to Algorithm 1 include the learning rate α, parameter ϵ (see Equation (20)), the number of learning rounds *n*, connection of all nodes Node_list, link status between all nodes Link_status, and the pair of the computing resource requester and the target computing node $\left(T_{src},T_{dst}\right)$. The output is the set of optimal reward routes for all node pairs given the link state. This path is formed by state-action pairs with the lowest values in the Q-table. Finally, based on the distribution of node computing power, the optimal path from the computing request source to the target computing node is determined as the final result of computing routing. This path is then installed in the switches as flow tables, facilitating the scheduling of computing resources through the network.

The RSCR routing algorithm can find the optimal path for all pairs of nodes in the given topology. For each pair of nodes (src,dst) in the Nodelist, routing learning can be performed following the steps in Algorithm 1, lines 1–17. First, initialize the Q-table to 0 (line 2), and then, the algorithm goes through episodes (line 3) until the state $S_{t}$ becomes the final state dst (lines 5–9). Starting with src as the initial state (line 4), assuming the next state is not the target state dst (line 5), the agent selects the action $A_{t}$ based on Equation (20) (line 6). The agent calculates the reward value according to Equation (18) (line 7), then updates the state using Equation (19) (line 8), and moves to the new state (line 9) until the episode concludes.

After the RL agent completes learning, the optimal path between src and dst can be calculated based on the Q-table, specifically the path with the minimum Q-value (line 12). If (src,dst) is the same as $\left(T_{src},T_{dst}\right)$, then this node pair corresponds to the computing resource requester and target computing node pair we are looking for, and the corresponding path is the optimal computing resource path (lines 14–15). Finally, the knowledge plane stores routing information for all node pairs in Paths, and the optimal computing resource path is specially marked. Subsequently, the flow installation module retrieves these paths and installs them in the routing table of switches.
**Algorithm 1:** SD-CFN Routing: RSCR 
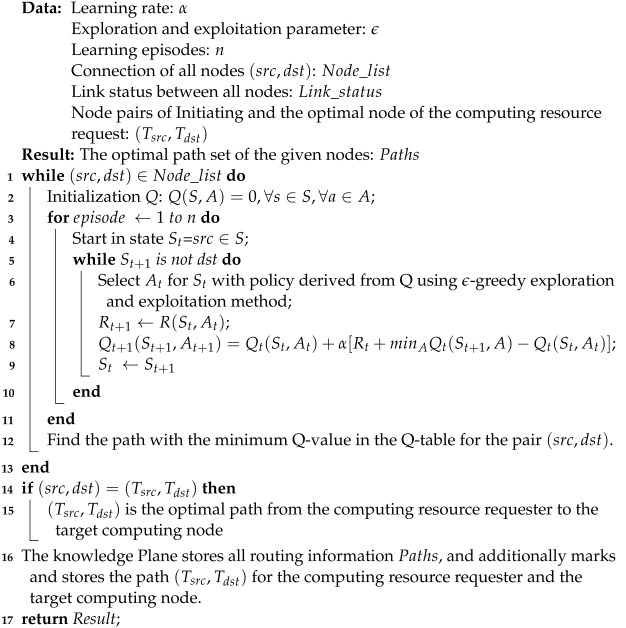


## 6. Results and Analysis

This section introduces the evaluation of RSCR. Section 6.1 describes the testing environment, Section 6.2 discusses signal generation, Section 6.3 introduces traffic generation, and Section 6.4 analyzes the experimental results.

### 6.1. Test Environment

**Figure 3:**
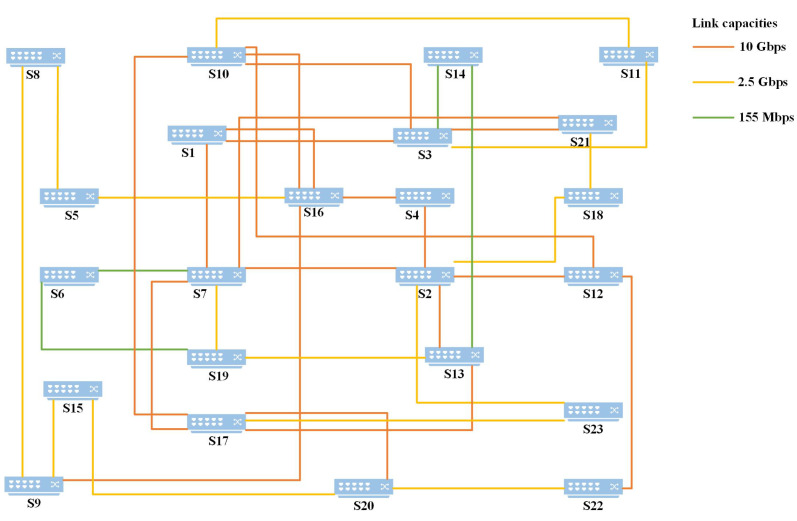
GÉANT topology.

Figure 3 shows the test topology of RSCR, namely the GÉANT topology. This topology is an international network in Europe used for education and research. GÉANT consists of 23 switches and 37 links, with 50% of the links at 10 Gbps, 40% at 2.5 Gbps, and 10% at 155 Mbps. We deployed the mentioned GÉANT topology in Mininet 2.3.1, with each switch connected to a host responsible for forwarding and receiving traffic. As Mininet’s virtual hosts cannot effectively provide real and valid computing resource information, the computing resource data were based on real-time data from a Kubernetes cluster.

The data plane of RSCR is composed of the GÉANT topology. In the control plane, a Ryu controller is deployed, and topology discovery, link-state perception, and flow table installation are all developed based on this controller. The management plane involves data processing using Python 3.9 with Pandas 1.5.3 and Numpy 1.24.3 libraries. The knowledge plane, developed using Python 3.9 and RL, is responsible for computing algorithmic routing decisions. We store link-state information in the CSV format and the computed routing results in the JSON format. The experimental environment is deployed on a system with an 11th Gen Intel(R) Core(TM) i7-1165G7 processor and 16GB RAM running Ubuntu Desktop 20.04.

### 6.2. Traffic Generation

iperf is a commonly used traffic generation tool in Mininet, and, accordingly, we have developed an iperf script to run on both the host and client sides. Following reference [48], the authors computed a traffic matrix for approximately 4 months at 15-minute intervals to conform to the XML format of TOTEM, providing the traffic matrix. We employ this traffic matrix to introduce background traffic to the GÉANT topology.

### 6.3. Parameters Setup

**Figure 4:**
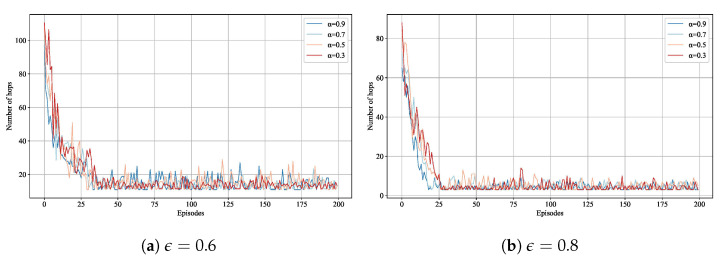
Number of Hops for Different Learning Parameters.

In RL algorithms, determining the appropriate values for the learning rate (α) and exploration rate (ϵ) is a crucial issue. To address this, we use the number of hops in the path between the source and destination addresses as a criterion. Initially, we run the RSCR routing algorithm, continuously adjusting α and ϵ to explore potential paths between Switch 2 and Switch 22. When link costs are equal, the shortest path found by Dijkstra’s algorithm is three hops. For each ϵ(0.8,0.6), we test four α values (0.9,0.7,0.5,0.3) for 200 iterations each time. Figure 4 illustrates the number of hops between Switch 2 and Switch 22 under different parameters. As observed in the figure, reducing ϵ leads the RL agent to explore random actions in the action space, increasing the number of hops. Conversely, the agent tends to leverage previous experiences when ϵ is high. For α=0.9 and α=0.7, it is evident that the convergence speed of α=0.9 is superior to that of α=0.7. Based on these results, the most suitable values for the parameters of the RL agent in RSCR are α=0.9 and ϵ=0.8.

### 6.4. Results and Analysis

**Figure 5:**
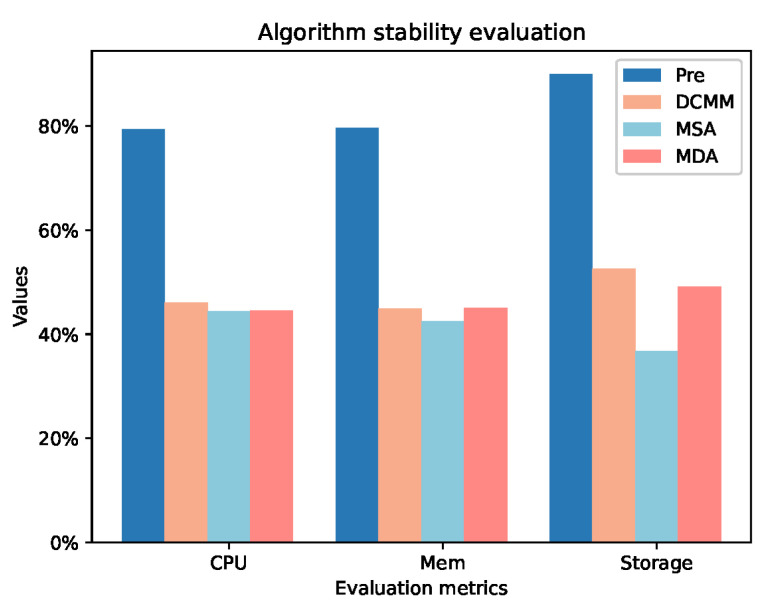
Algorithm Stability Assessment.

Figure 5 illustrates the impact of DCRMM, MDA, and MSA on the stability of computing nodes. We conducted tests on the average decrease rates in CPU, Memory (Mem), and Storage for all nodes after executing 10 tasks. The average decrease rate is defined by Equation (21), where $Node_{pre}$ represents the average utilization before task execution, and $Node_{now}$ represents the average utilization after task execution. The average CPU decrease rate of DCRMM is 1.60% lower than MSA and 1.45% lower than MDA. The average Mem decrease rate is 2.43% lower than MSA and 0.14% lower than MDA. For Storage, the average decrease rate is 15.80% lower than MSA and 3.48% lower than MDA. This indicates that DCRMM can reduce the variability of computational resources on nodes, enhancing stability among nodes.

(21) $$\begin{array}{c} S=Node_{pre}-Node_{now} \end{array}$$

**Figure 6:**
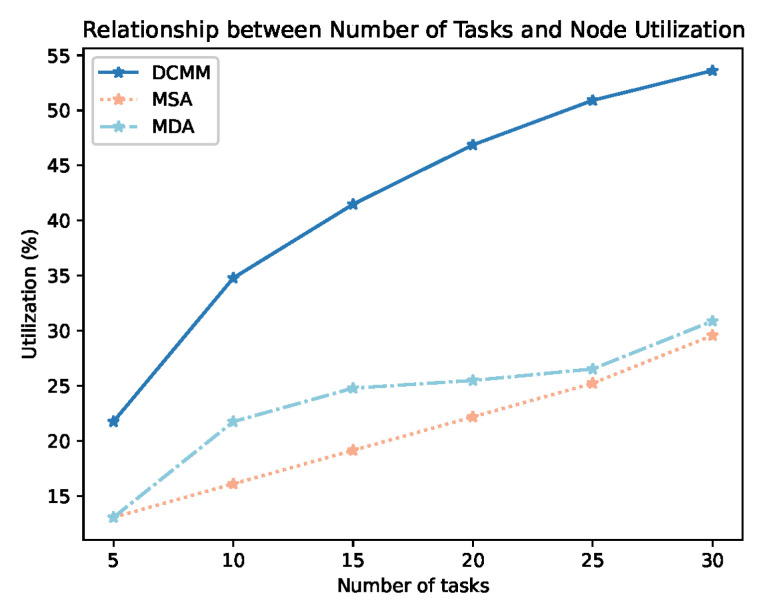
Relationship Between Node Utilization and Number of Tasks.

Figure 6 depicts the relationship between node utilization and the number of tasks for DCRMM, MDA, and MSA. As shown in Equation (22), node utilization (*U*) is represented as the ratio of selected nodes ($Node_{s}$) to the total number of nodes ($Node_{t}$). From Figure 6, it is evident that with an increase in the number of tasks, the node utilization of DCRMM, MDA, and MSA all experiences a corresponding improvement. However, it is clear from the figure that the node utilization of DCRMM is significantly better than that of MDA and MSA, both at low and high task numbers. This is attributed to the fact that DCRMM can simultaneously consider static and dynamic metrics, enhancing the precision of node-task matching.

(22) $$\begin{array}{c} U=\frac{Node_{s}}{Node_{t}} \end{array}$$

**Figure 7:**
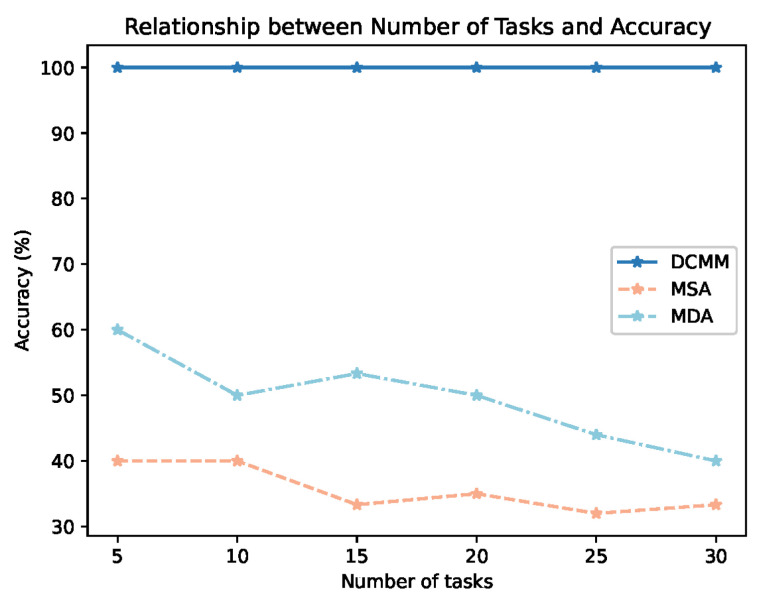
Relationship Between Node Accuracy and Number of Tasks.

Figure 7 represents the relationship between the matching accuracy of selected nodes by DCRMM, MDA, and MSA and the number of tasks. As indicated in Equation (23), the matching accuracy (*A*) is represented as the ratio of nodes meeting the requirements of tasks ($Node_{m}$) to the selected nodes ($Node_{s}$). According to the figure, the matching accuracy of DCRMM can be maintained at 100%, while MDA has a matching accuracy of only below 60%, and MSA is below 40%. Moreover, with an increase in the number of tasks, both MDA and MSA show a decline in matching accuracy to varying degrees. This is because the DCRMM algorithm considers both dynamic and static metrics, performs node matching by calculating Euclidean distance, and prioritizes the removal of nodes that do not meet the requirements before computing the Euclidean distance. This ensures that all nodes in the filtered pool can meet the computational requirements of the tasks.

(23) $$\begin{array}{c} A=\frac{Node_{m}}{Node_{s}} \end{array}$$

**Figure 8:**
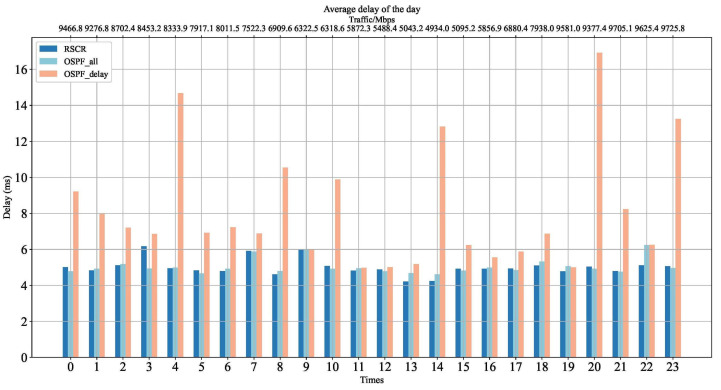
Average Delay Comparison Chart.

Figure 8 illustrates the average link delay of the RSCR. According to Figure 8, it can be observed that the average delay of the RSCR is comparable to that of the OSPF algorithm. However, when compared to the OSPF_delay algorithm, the average delay of the RSCR algorithm is lower by 63%. This is because the RSCR exhibits a preference for paths with fewer hops and lower congestion levels in route selection.

**Figure 9:**
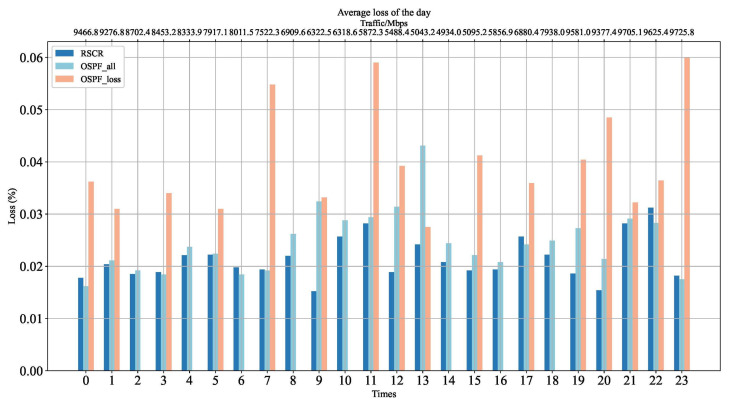
Average Packet Loss Rate Comparison Chart.

Figure 9 depicts the average packet loss rate of the RSCR algorithm. According to Figure 9, it is evident that the packet loss rate of RSCR is 15% and 25% lower than OSPF_all and OSPF_loss, respectively. In some scenarios, the packet loss rate of OSPF_all is more than twice that of the RSCR algorithm. This is attributed to the RSCR algorithm’s capability to select relatively shorter paths, thereby reducing packet loss.

**Figure 10:**
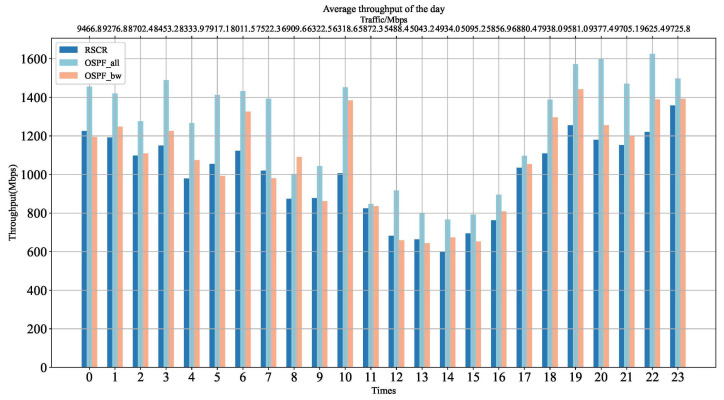
Average Throughput Comparison Chart.

Figure 10 illustrates the average link throughput of RSCR throughout the day. The throughput of RSCR is lower than OSPF_all and OSPF_bw, primarily because RSCR selects paths that are less preferred by the latter two, resulting in a larger denominator when calculating the average link throughput. This also explains why the average delay and average packet loss rate of RSCR outperform other algorithms, while the throughput remains at a lower level.

## 7. Conclusions and Future Work

This paper proposes a unified modeling and measurement method for the dynamic and static performance indicators of computing nodes, named DCRMM. The paper provides a detailed explanation of how to select the optimal computing nodes based on measurement results. Experimental results indicate that compared to computing node measurement methods that only consider dynamic or static indicators, DCRMM demonstrates superior node stability, node utilization, and node matching accuracy, enabling more effective utilization of node resources. Additionally, the paper discusses a computing power routing algorithm, RSCR, based on the SD-CFN architecture. This algorithm employs a Q-Learning mechanism to effectively compute paths between the computing power requester and the computing power provider. We validate the algorithm on the GÉANT topology, and the experiments show that RSCR can ensure a significant reduction in packet loss while maintaining a delay close to that of the OSPF algorithm. Furthermore, it increases link utilization, allowing traffic to be distributed more widely across the entire network topology.

Based on this, we hope that future work can explore the use of transfer learning algorithms for more experimentation on dynamic topologies. This would enable the algorithm to make new decisions quickly in situations where the topology is constantly changing.

## Data Availability

The code of this article is open source at https://github.com/ryan1016/RSCR/ (will be available online in March 2024).
